# A Practical Treatise on the Medical and Surgical Uses of Electricity

**Published:** 1883-11

**Authors:** 


**A Practical Treatise on the Medical and Surgical Uses of Electricity.** Including localized and general Faradazation, localized and central Faradazation, Franklinization, electrolyses and galvanic cautery. By GEORGE M. BEARD, M. D., and A. D. ROCKWELL. Fourth edition, revised by A. D. ROCKWELL, with nearly 200 illustrations. New York: Wm. Wood & Co., Publishers, 56 and 58 Lafayette Place. 1883.

This book is so well known that it is hardly necessary to do more than announce the appearance of a fourth edition. It has for years been the recognized guide in the department of which it treats, and to every physician who employs electricity in his practice (and who does not?) it is almost a necessity. Dr. Rockwell has, in many ways, improved the book by his last revision. It is now thoroughly up to the present knowledge in all the departments of its subject. Perhaps the most important of the new matter that has been introduced relates to the experience of the author in the treatment of extra uterine pregnancy. Formerly, as is well known, these cases resulted either in immediate death, through rupture of the distended tube, or in protracted suffering, with frequently a fatal ending, through the efforts of nature to rid iself of the fœtal mass. By the agency of electricity, in the method fully described in this edition, this abnormality of pregnancy may be met and treated with comparatively little danger or suffering to the mother. Extra uterine pregnancy is doubtless more frequent than is generally supposed. It is of the greatest importance that every practitioner be on the alert for this condition in its earlier stages, and familiar with a method of treatment which has proved efficacious. Messrs. Wood & Co. have published the book in excellent form. It contains nearly 800 pages, and the illustrations are excellent and sufficiently numerous.

